# Online longitudinal monitoring of brain health in former contact sport athletes: A study of acceptability and ethicality

**DOI:** 10.1002/ejsc.12052

**Published:** 2024-03-18

**Authors:** Ellen Frances Boucher, Michael J. Grey, Michael Hornberger, Sarah Hanson

**Affiliations:** ^1^ Faculty of Medicine and Health Sciences University of East Anglia Norwich UK; ^2^ School of Sport, Exercise and Health Sciences Loughborough University Loughborough UK

**Keywords:** assessment, cognition, ethics, health, methodology

## Abstract

Retrospective studies reveal that retired professional football players are at an increased risk of dementia. Prospective, longitudinal evidence in athletes diverse in sex, playing level, age and sport are now needed to understand the link between contact sport and dementia. The SCORES (Screening Cognitive Outcomes after Repetitive head impact Exposure in Sport) project investigates brain health online of former contact and non‐contact sport athletes as they age. Longitudinal study success is dependent on recruitment and retention. Both are influenced by perceived acceptability of participation. The SCORES project also offers regular feedback on assessment performance to participants, which raises ethical challenges. This study was designed to explore acceptability of the SCORES project to improve recruitment, retention and ensure ethicality amongst participants. Eight participants were purposively sampled and interviewed based on Sekhon's theoretical framework for assessing acceptability. Responses were analysed deductively against this framework. Main findings were that promoting altruistic and personal benefits of participation could aid recruitment. Conversely, computer literacy and the possibility of discovering a decline in their brain health was a potential barrier. Participants identified clarity of instructions, regular non‐intrusive researcher contact, low assessment burden, emphasis on participation as voluntary and the promotion of a community as avenues towards improving retention. They identified assessment frustration and challenging assessments as possible reasons for attrition. Participants viewed feedback as both necessary and important and made suggestions for ensuring ethicality. Findings from this study demonstrate how longitudinal online studies of athletes can be improved to aid recruitment, retention and ethicality.

## Highlights

- This study assessed the acceptability of an existing online longitudinal study protocol that monitors brain health in former athletes and provides feedback on performance in cognitive assessments.
- Online study environments reduce some of the burden associated with participation in online studies and enable recruitment of diverse athlete populations. Assessing acceptability for participants in online longitudinal health studies in an athlete population is important for improving protocols and efficiency of time and resources.
- This investigation demonstrates that participants find the online environment to be acceptable and makes suggestions for improving recruitment and retention. The inclusion of a feedback process is important for recruitment and retention, and participants outline a procedure that would be ethical and meaningful for them.
- Findings from this study can be used to inform design, improve recruitment and retention and integrate ethical feedback procedures into online longitudinal studies of athletes.

## 1 INTRODUCTION

Growing evidence suggests that former professional contact‐sport athletes are at a higher risk of dementia (Batty et al., 2023; Mackay et al., 2019). Previous investigations are mostly limited to studying male former professional athletes, although a small body of evidence suggests that amateur association footballers, wrestlers and boxers may be at an increased risk of dementia compared to the general population (Batty et al., 2023). Growing concern exists for females, who may be at a greater risk for sport‐related neurodegenerative disease than males (McGroarty et al., 2020; Sanderson, 2021). However, females are underrepresented in research to date (D’Lauro et al., 2022). Additionally, previous investigations have relied on health record data (Mackay et al., 2019) or data from a single measurement (Prien et al., 2020). Dementia and its cognitive, mental and behavioural symptoms are progressive and therefore investigations of the progression of these symptoms need to take repeated measures to monitor the long‐term effects of exposure to sport‐related head injury. Therefore, study designs need to cater for diverse recruitment, include study protocols that are accessible to participants from all levels of sport and support long‐term participation.

Whilst longitudinal studies offer valuable information on change over time, cause and effect relationships, and the sequence of outcomes, they are also expensive, take time to produce meaningful results and are time‐consuming for both researchers and participants. To produce meaningful results, large group sizes are needed to monitor group differences, monitor change over time and adjust for risk and preventative factors in analysis. The success of such studies therefore relies on effective recruitment and retention of participants.

In October 2020, the SCORES (Screening for Cognitive Outcomes after Repetitive head impact Exposure in Sport) project was launched to address these methodological challenges. The SCORES project is a longitudinal study designed to monitor the brain health of athletes as they age through online assessments every 3 months. This allows researchers to monitor for group differences and change over time that are meaningful for both the researcher and the participant. Participants include males and females from a range of contact or non‐contact sporting backgrounds (professional, amateur and recreational) as well as participants who report no participation in sport to create a range of comparisons. The assessments consist of traditional cognitive assessments translated to an online format. Following data collection for this study, a set of online self‐report questionnaires was also introduced to monitor mental health and behaviours. Assessment batteries last approximately 30 min. The online format was chosen to extend the recruitment reach, and to suit the needs of participants from all levels of sport.

The study design offers participants the chance to receive feedback on their performance in assessments, which they can take to a healthcare professional, with the aim of aiding early identification and intervention. Participants can opt for this process. This feedback summarises performance in cognitive assessments in comparison to other participants similar in sex and age in the SCORES sample and a normative sample. The feedback is not designed to provide a diagnosis of dementia, but to give participants an understanding of how they have performed in relation to people similar to them over time. Offering performance feedback potentially poses a number of ethical challenges, whereby the benefits of aiding early identification and personal understanding of brain health are counterbalanced by challenges in creating unnecessary anxiety and potential harm to participants. Exploring acceptability is therefore vital to promote avenues to ensure ethicality, improve recruitment and reduce attrition rates in a diverse participant group.

Sekhon defines acceptability as a multifaceted construct that reflects the extent to which people delivering or receiving a healthcare intervention consider it to be appropriate, based on anticipated or experiential cognitive and emotional responses to the intervention (Sekhon et al., 2017). Whilst the framework is typically applied to the assessment of interventions, previous studies have also assessed the acceptability of participation in longitudinal research (Kirkland et al., 2009). Assessing the prospective, concurrent and retrospective acceptability of longitudinal studies allows improvement of protocols to reduce the waste of time and data for the researcher and the participant. Therefore, this study aimed to investigate the acceptability of online participation in a longitudinal study that monitors the long‐term effects of repetitive sport‐related head injury in former athletes and provides feedback on brain health. The study was designed to explore the topics of recruitment, retention and ethicality of providing feedback on performance in a longitudinal study monitoring brain health.

## 2 MATERIALS AND METHODS

Ethical approval for this study was granted from the University of East Anglia Faculty of Medicine and Health Science Ethics Committee in August 2020 (Ref: 2019/20‐143). Semi‐structured interviews were considered to be the most appropriate form of data collection because they allowed the researcher to guide vdiscussion through a range of topics related to the design of the SCRES protocol and also allow participants to lead discussion about their experience of the project and perception of acceptability (King et al., 2019). Sekhon's acceptability framework (Sekhon et al., 2017) informed the development of questions around prospective, concurrent and retrospective acceptability of affective attitude, burden, ethicality, intervention coherence, opportunity costs, perceived effectiveness and self‐efficacy or participation. For example, questions included ‘What prompted you to get involved as a participant in the project?’ and ‘How do you feel about receiving feedback about your brain health?’. The topic guide is appended (Appendix A). The interviews were conducted by the first author, a female postgraduate researcher with a history of participation in amateur sport. Social biases were accounted for by the first author encouraging participants to answer openly and honestly because responses would be used to improve the study and feedback process and regular discussions with a co‐author during this stage.

Participants in the SCORES project were invited to complete a set of cognitive, mental health and behavioural assessments every 3 months for at least 10 years. Following each set of assessments, participants were invited to provide optional feedback about the study in short questionnaire, which informed the design of interview topics. All the participants who had completed their first set of online assessments (typically 3 months into participation), received information about this qualitative acceptability study and were invited to register their interest.

Participants had to be taking part in the SCORES project and therefore met the project inclusion criteria: aged over 40, lived in the UK at time of data collection and did not have a diagnosis of dementia. A purposive sample (*n* = 8) was chosen, from those who expressed interest, to provide a balance of age, sex and sporting history and all consented to take part (see Table 1). Purposive sampling by ethnicity was limited because all participants that registered their interest were white British.

**TABLE 1:** Participant characteristics describing key demographic information relevant to this study.

Participant number	Age	Sex	Sporting history
1	59	Male	Amateur Football
2	51	Male	Professional Football
3	56	Male	Amateur Football, Recreational Cycling and Recreational Snowsports
4	86	Female	Recreational Swimming
5	59	Female	Recreational Hockey and Recreational Netball
6	79	Male	Amateur Football, Amateur Athletics and Amateur Golf
7	59	Male	Amateur Football
8	75	Male	No Sporting History

*Note*: Participants were asked to report the top three sports that they participated in throughout their life and the highest level at which they played this sport.

Interviews took place over Zoom, lasted approximately 30 min and were transcribed by the first author. Data were analysed deductively against predetermined themes based on Sekhon's acceptability framework (Sekhon et al., 2017). This framework includes affective attitude, burden, ethicality, intervention coherence, opportunity costs, perceived effectiveness and self‐efficacy of participation.

## 3 FINDINGS

Following our deductive analysis, based on Sekhon's Acceptability Framework, responses from participants were organised into three topics: recruitment, retention and the ethicality of the feedback process.

### 3.1 Recruitment

To understand what attracted participants to volunteer their time, participants were asked to describe their original motivation. Some described an awareness of their own brain health as a motivator.

Having played for most of my life… as I saw the information I thought well, it’s worth knowing. My father passed away three or four years ago as well and he was sort of a keen amateur footballer and he had dementia in the end. So fairly close to my heart. (P1)

My view is that if we don’t volunteer, we can’t do this work, and we can’t all benefit. I’m of an age now, over 50, where stuff that’s going to happen in the next 20, 30 years will probably affect my health. That’s a slightly selfish aspect of it. (P3)

Interview volunteers described the opportunity to receive feedback on their brain health as a key motivation because of their history of sport or concussions, especially following high‐profile coverage of former athlete health.

I’ve sort of been interested with the link to dementia particularly after Jeff Astle and his death which was quite a while ago now. And there seem to be quite a few suffering from this. So, from my own personal perspective I am sort of interested in whether there were any sort of early warning signs or anything like that which might point to something for me. (P2)

I used to play amateur football and in my position as centre forward I used to head the ball a lot… But also, three and a half years ago I had a pretty bad cycling accident where I went through the back of a car, the back window, and I knocked myself out. I had a severe concussion. …So that concussion element of it for me was also interesting in terms of this particular project. (P3)

Participants also described an altruistic motivation towards volunteering.

To help in research and hopefully … help prevent (dementia) from happening through sports. To stop head injuries causing dementia. (P5)

It’s in the interest of mankind dare I say. (P6)

Participants identified key barriers to participation including affective attitude towards receiving poor feedback as well as the requirement for good computer literacy.

If anyone is concerned that they have suffered any neurological injury that it might highlight it and bring it home a little bit more than putting it in the back of your mind. (P1)

Only negatives I would say are for people to partake in this, if they’re not good with a computer they’ll struggle. (P6)

### 3.2 Retention

When asked about their expectations of the study, some participants reported having expected the testing to be more comprehensive, including a more detailed assessment of their neurological health, a physical examination or more in‐depth discussion of their sporting history.

I expected it to be a lot more intense. But it was a lot easier than I thought which made me feel a bit more relaxed. (P5)

I was expecting some sort of physical examination or physical test, and especially a discussion about my sort of activity as a footballer and heading the ball etc. (P3)

Assessments of mental health and behaviour were not originally included in the test battery to first explore their ethicality prior to their inclusion. At the time of recruitment, participants had an awareness of how the study format worked, and they could consider the acceptability of their inclusion. The participants revealed that assessment of mental health and behaviour were in line with their expectations of the study.

Personally I’m fine with it. Going into this with my eyes open I sort of anticipated that in some ways. (P1)

For most participants, the experience of online testing was positive. They described the experience as convenient and easy.

The fact that it’s not invasive, it’s not time consuming and with a lot of the online stuff you can do it from wherever. (P3)

The positive is that it is easy, and anyone can do it. (P5)

However, some participants identified that the online environment and cognitive assessments could be challenging.

I’ve not always found it easy to sign in and remember passwords or whatever, but actually doing the assessment—I quite enjoyed and I thought it was quite like a game really. (P6)

I found that the tests for me were too quick. (P7)

In addition to this, participants suggested that the frustration associated with making mistakes in the assessments could be a negative experience in participation, which could increase attrition.

The feeling of frustration when you’re making mistakes, but I think the positive is that you know why you’re doing it. (P3)

When asked how retention could be encouraged in the project, participants identified that the clarity of instructions were a key contributor to finding assessments easy and accessible.

I thought they were very good with the examples as well. …They were very clear and the way that they were spaced meant that I couldn’t read them too quickly. I had to read one, then see the example, then have a practice and then go on. (P3)

The regularity of contact was described as a positive aspect of participation, and participants expressed that the level of researcher contact could improve comfort in participation.

The contact I have had (has) not been intrusive at all. (P2)

If you keep people in the loop and keep people up to date then they will stay with you. If you go quiet that’s when people go they’ve lost interest in me. (P3)

Online I can think oh yeah that’s (the researcher), I can call her if I need to know anything or drop her a line. The friendliness is really helpful. It really makes a difference. (P5)

Participants also described the emphasis on participation as voluntary and the reminder that participants can opt out at any point is an important contributing factor for reducing burden and promoting participation.

I think reassuring people that if they do want to leave… that’s reassuring to know that it’s their choice. Some people sign up for something and then after a while they think no, I don’t want to do this anymore and then they feel guilty and pressured, and that you have given us the choice to leave if we want to. (P5)

Participants also discussed the importance of developing and emphasizing a community and the scale of the project in retaining participants.

Also some form of indication about how many people are taking part because I think again, being part of a community helps. It’s not just me, there are other people taking part. (P3)

### 3.3 Ethicality of feedback process

All participants interviewed in this study expressed an interest in receiving feedback on their assessment performance. The feedback process was identified as an opportunity for an external assessment of their brain health, and to give them insight into their brain health and the opportunity to act.

We can’t really know how good or bad our brain or how well our brain health is working apart from what we can tell ourselves, but it would be always useful to hear what somebody else generally thinks about it. Especially someone who has got a certain amount of sense about what they’re talking about. (P8)

Even if it was very negative news because I think that would enable me to start action. (P2)

Although participants highlighted the benefits of receiving feedback, one reflected concern around receiving negative or potentially distressful news.

If I got a poor score there, I’d be quite upset really. Not with you, but thinking I’ve got a problem and start to worry. (P6)

Participants were asked to describe a format that they found acceptable for receiving cognitive results. They described a preference for emailed written reports, which could be shared with their GP. Signposting to resources other than the GP were also desirable.

Well just an email saying you’ve got ill health and that you should see a medical practitioner. I don’t see that you need to give huge advice on it. (P7)

If they have a letter, I can take it to the doctor and if they think I am heading towards slight dementia or something like that then the doctor can take it from there. So, it is evidence as well as information. (P5)

If there were issues, I’d like to also be sent details of where I can get help or support. (P2)

Participants also identified that a visual representation of their performance and a reference point to compare their results to other participants who are similar to them would be helpful.

A graph would be good so you can see your ups and downs. (P5)

I think it needs to be age related, because people of 80 are going to be a little slower than people of 50. (P6)

The option to discuss results was also emphasised as important to the feedback process.

I guess a combination of written report and if it’s not good or it’s technical the opportunity to discuss. … I guess (with) someone involved in the study. Not necessarily an academic but maybe someone with experience of what’s being studied. Would they have to be medical—I'm not sure as long as I understood the results and could explain the results then that would be fine for me. (P1)

Participants also considered the ethicality of whether feedback should be provided for performance on measures of mental health and behaviour. They emphasised the importance of receiving feedback, particularly about mental health.

Some people don’t want to admit that they have got a problem, and it sometimes takes someone else … to say you need help go and get it. (P5)

If I thought that I had a mental health problem developing, and I wasn’t aware of that, I would probably be grateful if privately I was advised by somebody who knew what they were talking about. … Because people, men probably more than women, are always reluctant to get help. (P6)

I think it would be very useful for your organisation to mention to somebody if you see that they are beginning to suffer from any of these problems, because until a person knows the situation, they can’t do anything about it, and if they want to do something about it then it would be useful to have that information. (P8)

This discussion was balanced by participants revealing concerns about the outcomes of receiving feedback, particularly surrounding aggressive behaviour.

I think you’ve also got to think about…. if someone’s showing those (aggressive) tendencies to then receive something to say you’re test results are showing that those traits… how that could potentially affect people they live with or socialise with. (P2)

I think if you’re telling somebody that they’re showing signs of depression it might be a relief that people know. …. On the anger side I don’t know whether that could trigger something that you would probably want to make sure that the individual is in a safe environment. (P2)

## 4 DISCUSSION

This acceptability study explored participant motivation, experience and perception of ethicality of a longitudinal online study of athlete brain health that provides performance feedback to understand opportunities to improve recruitment, retention and ensure ethicality.

This investigation revealed that motivations to join the study included an awareness of participants' own brain health as a consequence of playing contact sport or a family history of dementia and the opportunity to receive feedback. Participants also expressed an altruistic motivation towards volunteering for the sake of protecting the next generation of athletes. The importance of altruistic motivations and affective attitude was also found in the acceptability study of the Canadian Longitudinal Study of Ageing (CLSA; Kirkland et al., 2009). Findings from the CLSA suggest that these motivations are important benefits of the study to highlight in recruitment. However, a basic need for computer literacy and concerns around receiving feedback were identified as potential deterrents to the study. This demonstrates a limitation of online studies in a study population that is diverse in age, education and socio‐economic status, and highlights the need for strong technical support in participation. Our findings also suggest that highlighting the opportunity to receive feedback is a good avenue for recruitment, but that participants want this to be an opt‐in procedure.

Benefits of participation included a low time burden and an assessment schedule that could be completed when convenient from their own home with a familiar computing device. The inclusion of mental health assessments in an online study format were deemed acceptable and were expected within the topic of monitoring brain health online. Participants found cognitive assessments to be mixed in terms of ease, and suggested that frustration could occur as a result. However, clarity of instructions and the opportunity to contact researchers by email were identified as helpful in reducing frustration and improving ease of online assessment. Participants highlighted that creating a community feeling also important to improving the long‐term retention of participation in the project. Interestingly participants also suggested that emphasising the voluntary nature of the project was important to retention, particularly that the consent form includes a statement about being able to drop out at any time, and that rather a mandatory time commitment might deter participants. After running for 2.5 years, the SCORES project has an attrition rate of approximately 20% of the total study population, which is lower than the estimates for other longitudinal studies, which ranges between 30% and 70% (Gustavson et al., 2012). It is yet to be determined whether the long‐term attrition rate is congruent with other longitudinal studies, but it could be suggested that in these early stages, the option to participate for as long as the participant wants as well as other previously mentioned factors, might increase retention at this stage.

The feedback process in the project is a key benefit of participation, particularly in a study that monitors for signs of dementia where early identification of prodromal symptoms are vital to understanding the disease and for providing early interventions (Rasmussen & Langerman, 2019). Receiving regular feedback was important to participants, not only as a measure of external assessment but also as a motivator to look after their brain health or seek the advice of a health professional. This is congruent with findings from the CLSA, which identified that the provision of individual results could catalyse long‐term involvement (Kirkland et al., 2009). The preferences for the format for this feedback were helpful in developing a procedure that was ethical and meaningful for participants. Participants wanted reports with peer comparisons that could be shared with a health professional or their family, the option for a discussion and signposting to relevant resources. Participants highlighted a need for sensitivity particularly when giving feedback about mental health and behaviour to reduce distress or propagation of behaviours. These findings suggest that inclusion of a feedback procedure within a longitudinal study is acceptable and beneficial to participants, but key considerations need to be made when designing feedback procedures. Feedback provision should be considered in terms of how it might affect the performance on future assessments, and the offer of discussions with team members needs to be considered in context of the scale of the sample size. However, this study suggests that inclusion of a participant feedback process that meets these needs is important for recruitment and retention, and has wider benefits for participants who can monitor their own brain health.

The diversity of participants that took part in this study was limited by ethnicity and sex, which are important factors in dementia risk research (Berry, 2015; Shiekh et al., 2021). Although the study sample is not reflective of society as a whole, it is reflective of those that initially volunteered for the study and the SCORES project. The project was advertised through multiple avenues but were limited by who actually volunteers. A strength of the study is the wide age range of participants, which offers insight into participation in online studies across the lifespan and with variation of computer literacy. Furthermore, the diversity of sport participation history offers helpful insights into participant motivation, particularly concerning health issues that may or may not directly affect them.

## 5 CONCLUSIONS

This study aimed to explore the acceptability of participation in an online longitudinal study that monitors the brain health of participants at regular intervals and provides feedback to participants. This investigation revealed that participants find the protocol and online environment of the study to be acceptable in the early stages, whilst also making helpful suggestions for improving recruitment and retention. The inclusion of a feedback process was identified as important for recruitment and retention, and participants made suggestions for a procedure that would be ethical and meaningful for participants. Findings from this study were used to directly improve the protocol of the SCORES project, and serve as recommendations for improving participant experience and reducing attrition in longitudinal online studies of athletes from all levels of sport.

## CONFLICT OF INTEREST STATEMENT

The authors report there are no competing interests to declare.

## APPENDIX: TOPIC GUIDE USED FOR SEMI‐STRUCTURED INTERVIEWS A

**Table:**

Topic	List of questions
Reason for participation	What prompted you to get involved as a participant in the project? How did you hear about the study? From how it was described, was it what you expected? (Prompt: If not, why not)
Experience in SCORES as a participant	4. Please describe your experience as a participant. Prompt: a. How did you find completing the online questionnaire? b. How did you get on with completing the brain health assessments? c. Was there an assessment that was particularly difficult/easy? In contrast, was there one that you found easier? d. Did you contact us for support in using the website, or completing any of the assessments and questionnaires? If so, how was the support that you received? Were you able to complete the tests OK after you got the support? 5. The feedback form revealed that many of our participants found the Sustained Attention to Response Task difficult (remind them which one). Was this the case for you? If yes, what made this test difficult to complete? 6. Would you recommend participating in this study to a friend? If yes, what would you say are the positives and negatives about taking part in this study? If no, why not?
Feasibility and acceptability	7. Having completed the first round of the project, how do you feel about completing further rounds of assessments and questionnaires in the future? 8. The feedback form revealed that most participants took roughly 30 min to complete the first round of the project. Future rounds of the project will be roughly the same length. How do you feel about this commitment of time? 9. The project intends to run for at least 10 years. Do you think participants would be able to commit to regular participation for at least 10 years? (Prompt: What would feel reasonable to you? What leads you to think that?) Prompt: a. What can we do to support that participation in the long term?
Receiving feedback about brain health	In the feedback form, we asked whether you would like to receive feedback about your brain health. We are currently in the process of making a decision about how to do this in a sensitive but helpful way. It is a bit tricky, as we know that many of our participants would like to receive feedback, but we need to work out how to do this in a way that is sensitive, and also in a way where we can deal with the situation where someone shows signs of poor brain health. So to help us to make this decision, we would like to ask you as a participant, how would you feel about receiving feedback about your brain health? 10. How do you feel about receiving feedback about your brain health? 11. How would you prefer to receive feedback about your brain health? 12. If a participant shows signs of poor brain health, how should this information be shared with them?
Mood and behaviour questionnaires	The project aims to study the brain health of people exposed to repetitive head injury. In some cases, people who are exposed to repetitive head injury may go on to develop a specific type of dementia called chronic traumatic encephalopathy (CTE). Not only does CTE effect brain health, but it can also lead to depression, anxiety, aggression or impulsivity. In the future, the project would like to measure these changes using specific questionnaires. 13. How do you feel about such questionnaires asking about your mental health or behaviour? 14. How would you feel about answering questions about your mental health in the future? 15. If a participant is showing signs of depression or anxiety, how should this information be communicated with them? 16. How would you feel about answering questions about aggressive or impulsive behaviour? 17. If a participant is showing high levels of aggression, how should this information be communicated with them? 18. If a participant is showing high levels of impulsivity, how should this information be communicated with them?
